# Evaluation of a Novel Plasmid for Simultaneous Gene Electrotransfer-Mediated Silencing of CD105 and CD146 in Combination with Irradiation

**DOI:** 10.3390/ijms22063069

**Published:** 2021-03-17

**Authors:** Monika Savarin, Urska Kamensek, Katarina Znidar, Vesna Todorovic, Gregor Sersa, Maja Cemazar

**Affiliations:** 1 Department of Experimental Oncology, Institute of Oncology Ljubljana, 1000 Ljubljana, Slovenia; ukamensek@onko-i.si (U.K.); kznidar@onko-i.si (K.Z.); vtodorovic@onko-i.si (V.T.); gsersa@onko-i.si (G.S.); 2 Biotechnical Faculty, University of Ljubljana, 1000 Ljubljana, Slovenia; 3 Faculty of Health Sciences, University of Ljubljana, 1000 Ljubljana, Slovenia; 4 Faculty of Health Sciences, University of Primorska, 6310 Izola, Slovenia

**Keywords:** CD105, CD146, plasmid, gene electrotransfer, antibiotic-free, irradiation

## Abstract

Targeting tumor vasculature through specific endothelial cell markers represents a promising approach for cancer treatment. Here our aim was to construct an antibiotic resistance gene-free plasmid encoding shRNAs to simultaneously target two endothelial cell markers, CD105 and CD146, and to test its functionality and therapeutic potential in vitro when delivered by gene electrotransfer (GET) and combined with irradiation (IR). Functionality of the plasmid was evaluated by determining the silencing of the targeted genes using qRT-PCR. Antiproliferative and antiangiogenic effects were determined by the cytotoxicity assay tube formation assay and wound healing assay in murine endothelial cells 2H-11. The functionality of the plasmid construct was also evaluated in malignant melanoma tumor cell line B16F10. Additionally, potential activation of immune response was measured by induction of DNA sensor STING and proinflammatory cytokines by qRT-PCR in endothelial cells 2H-11. We demonstrated that the plasmid construction was successful and can efficiently silence the expression of the two targeted genes. As a consequence of silencing, reduced migration rate and angiogenic potential was confirmed in 2H-11 endothelial cells. Furthermore, induction of DNA sensor STING and proinflammatory cytokines were determined, which could add to the therapeutic effectiveness when used in vivo. To conclude, we successfully constructed a novel plasmid DNA with two shRNAs, which holds a great promise for further in vivo testing.

## 1. Introduction

Targeting tumor vasculature through specific endothelial cell markers represents a promising approach for cancer treatment [1,2]. One of the noticeable targets of tumor vasculature is CD105 (Endoglin), a transforming growth factor β (TGF-β) coreceptor present on the surface of endothelial cells. In the normal conditions its expression is low, however upon activation of neoangiogenesis that is necessary for the progression of tumors, it becomes overexpressed, leading to changed regulation of migration, proliferation, differentiation and adhesion of endothelial cells [3,4,5,6]. Another tentative target of tumor vasculature is CD146 (melanoma cell adhesion molecule, MCAM), an adhesion molecule present on many tumors and on vascular endothelial and smooth muscle cells. CD146 is also known as VEGF-R2 coreceptor and activates nuclear factor NF-κB in endothelial cells, which in turn promotes transcription of many proangiogenic factors [7,8,9]. Attempts to downregulate either CD105 or CD146 have been made in several in vitro and in vivo studies using either specific antibodies or RNA interference [10,11,12,13,14,15,16,17].

In our studies, we showed that successful downregulation of either of these two markers can be achieved by a non-viral gene therapy approach called gene electrotransfer (GET) [10,16]. In this safe and efficient non-viral gene delivery approach, the permeability of cell membrane is increased after electroporation of cells or tissues, which enables the introduction of different nucleic acids. Several different electroporation systems exist for delivery of genetic material, but the working principle is the same [18,19,20,21,22]. In the case of pDNA, after application of controlled electric pulses to cells or tissues, a pDNA-membrane complex is formed on the membrane facing the cathode, then this complex enters the cells via endocytosis [23,24,25,26]. pDNA then escapes intracellular vesicles. The entry of pDNA to nucleus is still not completely understood, but it is not due to the passive diffusion across the nuclear envelope. Therefore, the limitation of the GET approach is that it is predominantly suitable for transfection of dividing cells, where pDNA can benefit from destabilization of nuclear envelope during mitosis to enter the cell’s nucleus. Namely, it was shown that synchronization of the GET with the mitotic phase increases the transfection efficiency. Nevertheless, it was demonstrated that only a small number of pDNA molecules, in the order of 10, is needed in the nucleus for transgene expression [27,28,29,30]. GET has already reached clinical applications for the purpose of cancer therapy, cancer vaccines or infectious disease vaccines [31,32]. In oncology, several clinical trials are ongoing for several types of cancer using pDNA encoding different therapeutic molecules, either immunomodulatory molecules such as interleukin 12 [33] or tumor associated antigens [34,35,36].

Plasmids, encoding different genetic information, can be designed to either overexpress a therapeutic transgene or silence an overexpressing gene through RNA interference [37]. Namely, therapy with siRNA was proven too short-lived for a good antitumor effect due to the instability of siRNA molecules. This drawback of siRNA can be alleviated by chemical modification of siRNA and by utilization of different delivery methods, such as nanoparticles or liposomes [38,39]. On the other hand, although pDNA needs to be delivered intranuclearly, studies have shown that the utilization of vector-based shRNA allows a longer and more stable expression of shRNA [39,40].

Additionally, compared to RNA, plasmid DNA is easier to produce and more stable and resistant to nucleolytic degradation. An important advantage of plasmid vectors is that the plasmid DNAs are capable of accommodating large genetic payloads, allowing cotransfection of multiple plasmids or transfection of larger polycistronic plasmids [41,42].

On the other hand, one possible limitation of plasmid DNA is the antibiotic resistance gene present in the backbone of conventional plasmids that is needed for their production bacteria [37]. Namely, antibiotics used in the production of such plasmids could be unsafe to patients that are allergic to antibiotic; furthermore, the antibiotic resistance genes could be transferred horizontally to environmental bacteria, making them resistant to antibiotics [43]. However, this can currently be circumvented by using various new technologies for preparation of antibiotic-free plasmids [44,45]. One of them is operator repressor titration (ORT) technology, which we have been successfully implementing in last couple of years to produce our plasmids [46,47,48].

Using GET of plasmids encoding shRNA against either CD105 or CD146, we have shown that these treatments can result in significant reduction of vasculature and good antitumor effectiveness in several tumor models [11,13,14,15,49,50]. The therapeutic effectiveness was even further improved when GET was combined with radiotherapy [14,15,51]. The rationale behind combining vascular targeted treatments with radiotherapy is that better oxygenation status of tumors can be achieved through normalization of the tumor vasculature, which than promotes tumor radiosensibilization [52,53,54,55].

In studies combining GET and radiotherapy, we also noticed that combined therapy activates the immune system, since more than half of the mice remained complete responders after a secondary tumor challenge [15,51]. To date, the immune-modulatory properties of both radiotherapy and GET are well documented. Stimulation of the immune response occurs even after GET of empty plasmids devoid of the therapeutic gene [56]. The explanation is that foreign plasmids DNA introduced in cells for gene-therapy and DNA fragments released from the radiotherapy-damaged tumor cells, act as pathogen associated molecular pattern (PAMPS) and danger associated molecular patterns (DAMPs) activating DNA sensors [56,57,58,59,60,61,62]. In mammalian cells, an important DNA sensor is the stimulator of interferon genes (STINGs) protein that triggers type I interferons and proinflammatory cytokine production. In our previous studies, we have shown activation of sensors after GET and also after IR separately, however we have not proven it after our combined treatment with GET of shRNA and IR [56,57].

Therefore, in the current study, our aim was to construct a plasmid that simultaneously targets both CD105 and CD146 and is devoid of the antibiotic resistance gene, and then to evaluate its functionally and therapeutic potential in vitro when delivered by GET and combined with irradiation (IR). Functionality of the plasmid was evaluated by determining the silencing of the targeted genes using qRT-PCR. Antiproliferative and antiangiogenic effects were determined by cytotoxicity assay, tube formation assay and wound healing assay in murine endothelial cells 2H-11. Additionally, functionality tests were performed in malignant melanoma tumor cell line B16F10. Potential activation of immune response was measured by induction of DNA sensor STING and proinflammatory cytokines by qRT-PCR.

## 2. Results

### 2.1. Construction of Plasmids

**Figure 1:**
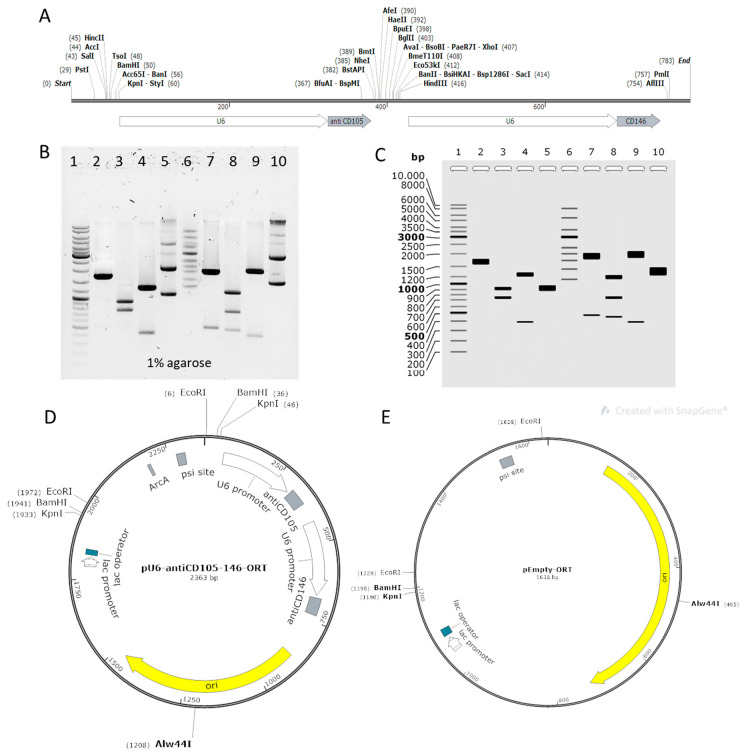
Construction of plasmids. (**A**) Assembly of the expression cassette encoding CD105 and CD146 shRNAs, each under its own U6 promoter. (**B**) Electrophoresis on 1% agarose gel stained with SybrSafe and run 1 h in TAE buffer at 100 V. (**C**) Restriction analysis simulation based on predicted sequence. Lane 1: GeneRuler™ DNA Ladder Mix; lane 2: pEmpty-ORT, KpnI: 1638 bp; lane 3: pEmpty-ORT, BamHI + Alw44I: 905 bp, 733 bp; lane 4: pEmpty-ORT, EcoRI: 1241 bp397 bp, lane 5: pEmpty-ORT, supercoiled: 1638 bp; lane 6: Supercoiled DNA Ladder; lane 7: pU6-antiCD105-146-ORT, KpnI: 1887 bp, 476 bp; lane 8: pU6-antiCD\1-\2-ORT, BamHI + Alw44I: 1172 bp, 733 bp, 458 bp; lane 9: pU6-antiCD105-146-ORT, EcoRI: 1966 bp, 397 bp; lane 10: pU6-antiCD105-146, supercoiled: 2363 bp. (**D**) pU6-antiCD105-146-ORT plasmid map. Full sequence is given in Supplementary sequence S1. (**E**) pEmpty-ORT plasmid map. Full sequence is Supplementary sequence S2. Simulation and maps were created in SnapGene software.

The dual targeting plasmid was constructed in two phases: first, the expression cassette encoding shRNA against CD105 and CD146 was assembled using artificial gene synthesis (Figure 1A), and then an antibiotic free version of the plasmid was prepared using ORT technology. Additionally, a plasmid devoid of expression cassette was constructed to serve as a control plasmid. The therapeutic recombinant plasmid encoding CD105 and CD146 shRNAs was named pU6-antiCD105-CD146-ORT and the empty control plasmid was named pEmpty-ORT. The successful construction of both plasmids was confirmed by restriction analysis. The plasmids were cut with different combinations of restriction enzymes and the identity of the plasmid was confirmed by positive matching of the pattern of bands on the electrophoresis gel to the expected pattern obtained by a simulation experiment using SnapGene software (Figure 1B,C) Finally, both plasmids were confirmed by full length plasmid sequencing and annotated plasmids maps were created based on sequencing results (Figure 1D,E). Additionally, the therapeutic plasmid was checked for presence of CpG islands and was verified CpG-free. The sequuences are available in supplementary data (Supplementary sequence S1 contains pU6-antiCD105-146-ORT full sequence, Supplementary sequence S2 describes pEmpty-ORT full sequence).

### 2.2. CD105 and CD146 Silencing

**Figure 2:**
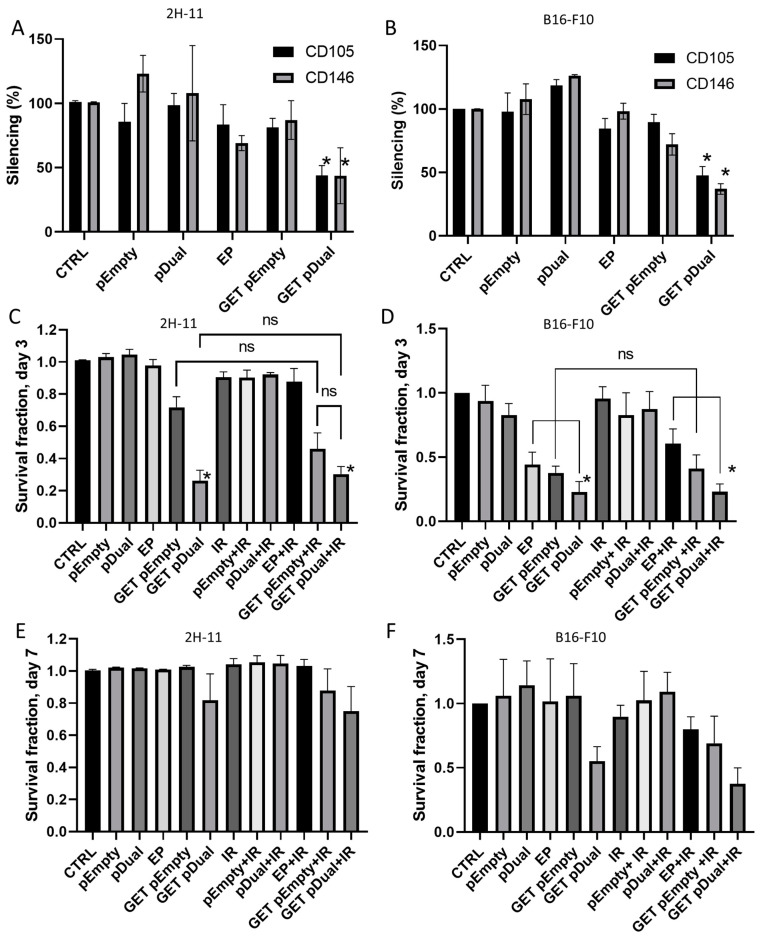
Silencing and cytotoxicity of pU6-anti-CD105-146-ORT GET. (**A**) Silencing of CD105 and CD146 after GET of pU6-anti-CD105-146-ORT plasmid in 2H-11 cells and (**B**) in B16-F10 cells. *, unpaired t test vs. CTRL (*p* < 0.05). (**C**) Cell survival after GET of pU6-anti-CD105-146-ORT plasmid alone or combined with IR on day 3 in 2H-11 cells and in (**D**) B16-F10 cells. (**E**) Cell survival after GET of pU6-anti-CD105-146-ORT plasmid alone or combined with IR on day 7 in 2H-11 cells and in (**F**) B16-F10 cells. Bars represent the mean ± standard error of mean for three independent experiments. Values are normalized to the control group. *, statistically significant difference vs. all groups (*p* < 0.05). ns, statistically non-significant difference. Group names: CTRL—untreated cells, pEmpty—treatment with pEmpty-ORT plasmid alone, pDual—treatment with pU6-anti-CD105-146-ORT plasmid alone, EP—application of electric pulses alone, GET pEmpty—GET of pEmpty-ORT plasmid, GET pDual—GET of pU6-anti-CD105-146-ORT plasmid, IR—IR with 5 Gy, EP + IR—application EP and IR with 5 Gy, GET pEmpty + IR—GET of pEmpty-ORT plasmid and IR with 5 Gy, pDual + IR—GET of pU6-anti-CD105-146-ORT plasmid and IR with 5 Gy.

The silencing ability of pU6-anti-CD105-146-ORT plasmid was tested after GET in the mouse endothelial cell line 2H-11 and melanoma cell line B16-F10 that was selected for the functionality test. Before the silencing experiment, we checked if both markers targeted by our new plasmid pU6-anti-CD105-146-ORT are expressed in 2H-11 and B16-F10 cells. The qRT-PCR results confirmed the expression of CD105 and CD146 in 2H-11 cells and B16-F10 cells: in the 2H-11 cell line average threshold cycle value (Ct) for CD105 was 26 and for CD146 25, and in B16-F10 cells Ct for both CD105 and CD146 was 25. After pU6-anti-CD105-146-ORT GET (GET pDual) both markers were significantly silenced compared to untreated control group (CTRL) in both cell lines. CD105 was silenced to 46% in 2H-11 cells and to 52% in B16-F10 cells, and CD146 to 51% in 2H-11 cells and to 63% in B16-F10 cells (Figure 2A,B).

### 2.3. Cytotoxicity

Cell survival after pU6-anti-CD105-146-ORT GET alone or combined with IR was measured on day 3 (Figure 2C,D) and day 7 (Figure 2E,F) in B16-F10 and 2H-11 cell line. GET of the therapeutic plasmid alone (GET pDual) or in combination with IR (GET pDual + IR) significantly decreased cell survival on day 3 compared to complete control (CTRL) and pertinent control groups (pEmpty, pDual, EP, IR, pEmpty + IR, pDual + IR and EP + IR) in both cell lines. Reduction of cell survival was evident also after GET of the control plasmid alone (GET pEmpty) or in combination with IR (GET pEmpty + IR). At day 7 the differences diminished, indicating on cell repopulation.

### 2.4. Inhibition of Capillary Tube Formation

**Figure 3:**
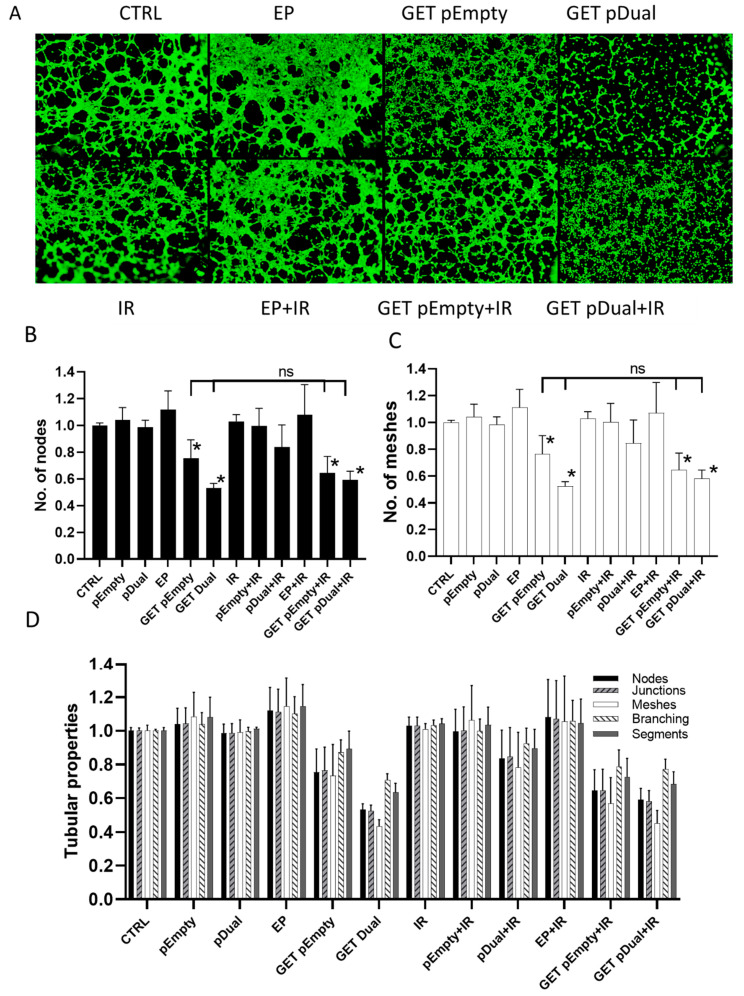
The effect of pU6-anti-CD105-146-ORT gene GET alone or in combination with IR on the capillary tube formation. (**A**) Representative images of 2H-11 tubular complexes after pU6-anti-CD105-146-ORT GET and IR. (**B**) Number of 2H-11 nodes after pU6-anti-CD105-146-ORT GET and IR. (**C**) Number of 2H-11 meshes after pU6-anti-CD105-146-ORT GET and IR. (**D**) Various 2H-11 tubular properties after pU6-anti-CD105-146-ORT GET and IR. Bars represent the mean ± standard error of mean for three independent experiments. Values are normalized to the control group. *, statistically significant difference vs. all groups (*p* < 0.05). ns, statistically non-significant difference. Scale bar = 500 µm. Group names: CTRL—untreated cells, pEmpty—treatment with pEmpty-ORT plasmid alone, pDual—treatment with pU6-anti-CD105-146-ORT plasmid alone, EP—application of electric pulses alone, GET pEmpty—GET of pEmpty-ORT plasmid, GET pDual—GET of pU6-anti-CD105-146-ORT plasmid, IR—IR with 5 Gy, EP + IR—application EP and IR with 5 Gy, GET pEmpty + IR—GET of pEmpty-ORT plasmid and IR with 5 Gy, pDual + IR—GET of pU6-anti-CD105-146-ORT plasmid and IR with 5 Gy.

Cell tube formation assay showed reduced 2H-11 endothelial cells tube formation after GET of the therapeutic plasmids, with or without IR (Figure 3A). The image analysis confirmed statistically significant reduction in the number of nodes (Figure 3B) and meshes (Figure 3C) after GET of the therapeutic plasmid alone (GET pDual) or in combination with IR (GET pDual + IR.) Reduction in the number of junctions and total branching and segments length after GET was also indicated, but did not reach statistical significance (Figure 3D).

### 2.5. Inhibition of Endothelial Cell Migration

**Figure 4:**
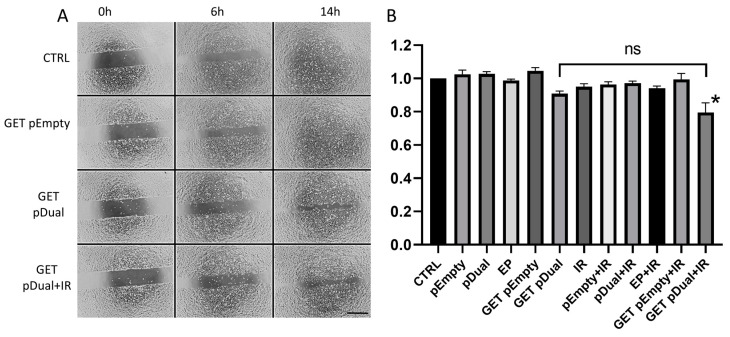
The effect of pU6-anti-CD105-146-ORT GET alone or in combination with IR on endothelial cell migration. (**A**) Representative images 2H-11 gap filling 6 and 14 h after pU6-anti-CD105-146-ORT GET and IR. (**B**) Results of the kinetic analysis of 2H-11 cell migration after pU6-anti-CD105-146-ORT GET and IR. Bars represent the mean ± standard error of mean for three independent experiments. Values are normalized to the control group. *, statistically significant difference vs. all groups (*p* < 0.05). ns, statistically non-significant difference. Scale bar = 500 µm. Group names: CTRL—untreated cells, pEmpty—treatment with pEmpty-ORT plasmid alone, pDual—treatment with pU6-anti-CD105-146-ORT plasmid alone, EP—application of electric pulses alone, GET pEmpty—GET of pEmpty-ORT plasmid, GET pDual—GET of pU6-anti-CD105-146-ORT plasmid, IR—IR with 5 Gy, EP + IR—application EP and IR with 5 Gy, GET pEmpty + IR—GET of pEmpty-ORT plasmid and IR with 5 Gy, pDual + IR—GET of pU6-anti-CD105-146-ORT plasmid and IR with 5 Gy.

The wound healing assay showed inhibition of endothelial cell migration after pU6-anti-CD105-146-ORT GET and IR (Figure 4A). After combined treatment with therapeutic plasmid GET and IR (GET pDual + IR), inhibition was statistically significant compared to all other groups except GET of the therapeutic plasmid alone (GET pDual) (Figure 4B).

### 2.6. Induction of Cytokine and STING Expression

**Figure 5:**
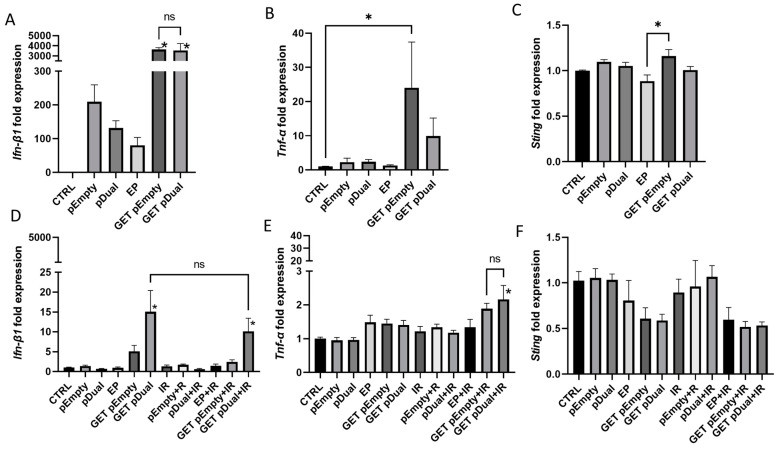
Expression of cytokines *Ifn-β1* and *Tnf-α* and DNA sensor *Sting* after pU6-anti-CD105-146-ORT GET alone or in combination with IR. Expression of *Ifn-β1* (**A**), *Tnf-α* (**B**) and *Sting* (**C**) 4 h after pU6-anti-CD105-146-ORT GET. Expression of *Ifn-β1* (**D**), *Tnf-α* (**E**) and *Sting* (**F**) 48 h after pU6-anti-CD105-146-ORT GET and 24 h after IR. Bars represent the mean ± standard error of mean for three independent experiments. Values are normalized to the control group. *, statistically significant difference vs. all groups (*p* < 0.05). ns, statistically non-significant difference. Group names: CTRL—untreated cells, pEmpty—treatment with pEmpty-ORT plasmid alone, pDual—treatment with pU6-anti-CD105-146-ORT plasmid alone, EP—application of electric pulses alone, GET pEmpty—GET of pEmpty plasmid, GET pDual—GET of pU6-anti-CD105-146-ORT plasmid, IR—IR with 5 Gy, EP + IR—application EP and IR with 5 Gy, GET pEmpty + IR—GET of pEmpty plasmid and IR with 5 Gy, pDual + IR—GET of pU6-anti-CD105-146-ORT plasmid and IR with 5 Gy.

Induction of expression of cytokines *Il1β*, *Ifn-β1* and *Tnf-α* and an DNA sensor *Sting* was measured 4 h after GET of pU6-anti-CD105-146-ORT, and then again 48 h later, i.e., 24 h after combination with IR. Expression of *Il1β* was not detected in 2H-11 cells, while, control Ct values for *Ifn-β1* were 33–34, 25–26 for *Tnf-α* and 21–22 for *Sting*, indicating high intrinsic expression of *Sting* and *Tnf-α* and low intrinsic expression of *Ifn-β1* in this cell line. A 4000-fold increase in expression of proinflammatory cytokine *Ifn-β1* was observed 4 h after GET of both therapeutic (GET pDual) and control plasmid (GET pEmpty) (Figure 5A). *Tnf-α* was also statistically significantly increased (20-fold) after GET of the control plasmid (GET pEmpty) (Figure 5B). The expression of DNA sensor *Sting* 4 h after GET was not statistically significantly increased compared to control group (Figure 5C), the only statistically significant difference was between the group receiving GET of the control plasmid (GET pEmpty) and group receiving application of electric pulses alone (EP).

After two days, the initial 4000-fold increase in *Ifn-β1*, fell down to 15-fold after GET of the therapeutic plasmids (GET pDual), which was still statistically significant compared to all groups, except the group receiving combination of therapeutic plasmid GET and IR (GET Dual + IR), where the increase was 10-fold (Figure 5D). *Tnf-α* was statistically significantly increased after combination of the therapeutic plasmid GET and IR (GET Dual + IR) (Figure 5E). There was no increase in *Sting* expression at this time point; just the opposite, its expression was even reduced, but not statistically significantly (Figure 5F).

## 3. Discussion

In this study, we described the construction of a plasmid devoid of antibiotic resistance gene that simultaneously targets two different signaling pathways, one TGF-β dependent, by silencing CD105 expression, and the other VEGFR2 dependent, by silencing CD146 expression. To our best knowledge, this is the first study describing and testing a dual vascular-targeting plasmid lacking an antibiotic resistance gene. The plasmid’s functionality and therapeutic potential was tested in vitro after delivery by GET in mouse endothelial cells 2H-11 and in combination with IR. Plasmid’s functionality was firstly confirmed by efficient silencing of both targeted genes. Additionally, in vitro testing has confirmed its antiangiogenic potential on endothelial cell line 2H-11, by reduced tube formation and migration. Finally, induction of DNA sensor STING and proinflammatory cytokines were determined, which could contribute to the therapeutic effectiveness when used in vivo.

The new plasmid was named pU6-anti-CD105-146-ORT and is intended for the use in GET, which allows transfer of multiple plasmids [41,63,64,65,66]. However, in case of shRNA coding sequences, which are relatively short (i.e., 55 base pairs) compared to gene coding regions, we decided that transfection of one polycistonic plasmid was more rational. Namely, the resulting bicistronic expression cassette was still small enough (550 base pairs) to support efficient transfection [67]. Based on the study of Mcintyre et al. [68], we decided to prepare a dual targeting plasmid with each shRNA sequence under its own promotor. The expression cassette of our new plasmid was assembled by combining the shRNA coding sequences specific for CD105 and CD146 mRNA that were selected in our previous studies, where high-rate silencing was achieved. The shRNA sequences were selected based on rationally designed siRNA sequences, which were also tested in silencing experiments [10,11,12,16]. Based on good experience with U6 promotor used in original plasmids, we kept the U6 promoter also in our new plasmid [69]. The expression cassette was than cloned in an antibiotic resistance-free backbone to ensure the safety of the patient and the environment, i.e., prevention of the possible horizontal gene transfer to commensal bacteria [7].

After the plasmid construction was confirmed, we evaluated its functionality by testing its ability to silence both CD105 and CD146. Using a qRT-PCR tests, we firstly demonstrated that 2H-11 cell line expresses both targeted genes, making it appropriate model for plasmid testing. After GET, we showed that our plasmid efficiently silenced both of the targeted markers for nearly 50%. These results are in accordance with our previous studies, where silencing was evaluated after GET of a single, non-antibiotic free plasmid, encoding either for anti CD105 (pENTR/U6 CD105) or anti CD146 (pMCAM) shRNA, where reduction of CD105 was for 57% [11] and CD146 for 72% [16].

Before proceeding with the further in vitro tests, we evaluated the cytotoxicity pU6-antiCD105-146-ORT GET alone or in combination with IR. The viability of the cells was checked on day 3 and 7 after GET. Our results indicate that on day 3, the viability of the cells was reduced after GET of the therapeutic pU6-anti-CD105-146-ORT plasmid, regardless of it was combined with IR or not. However, reduction was not statistically significant compared to the GET of a control pEmpty-ORT plasmid. Until day 7, the viability was restored in all groups, indicating on cell repopulation in this short time. These findings are in accordance with our previous studies, where reduced cell proliferation with either of the single plasmid was used [11,13,16] suggesting that dual plasmid retained, besides the ability of silencing, also cytotoxicity.

Due to the silencing of two antiangiogenic markers in endothelial cells, several biological properties, such as migration and tube formation, were affected after GET of our new plasmid. Migration levels were statistically significantly reduced only after pU6-anti-CD105-146-ORT GET combined with IR and not after GET of pU6-anti-CD105-146-ORT alone. The level of reduction in migration is not comparable to our previous studies with single targeting plasmids, since different cells or migration assays were used: For instance, in the studies using a CD105 targeting plasmid, xCELLigence real time analyzer was used to assess the inhibition of migration [11] or migration was evaluated on SVEC4–10 endothelial cell line [13]. While in the study using a CD146 targeting plasmid, the migration was assessed in the B16-F10 tumor cells [16]. In the current study, pU6-antiCD105-146-ORT GET with or without IR also hindered proper tube formation. The ability to form tubular complexes is an in vitro method indicating on a substances ability to interfere with angiogenesis. Similar findings were also demonstrated in other studies using single shRNA encoding plasmids [11,13,16,50], siRNAs [10] and oligonucleotides [70].

In order to predict the immunological effect, GET of our new plasmid could have when used in vivo, we evaluated distinct DNA sensing pathways. The results showed increased expression of *Ifn-β1* and *Tnf-α* after GET of both therapeutic and empty control plasmid, which indicates on the activation of the DNA sensing pathways. This effect is mediated by the presence of plasmid DNA in the cytosol and the accumulation of DNA in cytosol after IR, which activates several DNA sensing pathways in immune, tumor and non-tumor cells [56,57,58,71,72,73]. Even though IR alone can trigger DNA sensing pathways by DNA accumulation [57], the increased expression of cytokines after IR was not observed in our current study, probably due to the too low dose of IR (i.e., 5 Gy). The dose was selected based on our previous studies [14] and literature data [74,75,76]. Namely, to observed synergistic or additive effects of the treatments, IR alone should affect cell viability. However, in the present study, the IR alone did lead to expected reduction of cells’ survival of cells, which is the major limitation of our study. This is probably also the main reason for other less pronounced effects on migration and immunological features [76]. Namely, here we have also measured the expression of *Sting* in 2H-11 cells due to the fact, that cGAS-STING pathway was recognized as the main mediator of DNA sensing pathway in radiotherapy, leading to interferon (IFN) production [58]. The obtained results can therefore help to explain the results of our in vivo studies, where silencing of CD105 or CD146 was combined with IR, which potentiated the antitumor effectiveness resulting in the better therapeutic outcome on melanoma and carcinoma tumor models [15,51]. Therefore, to further elucidate the effectiveness of our new plasmid, it should be also tested in vivo.

To conclude, we successfully constructed a plasmid encoding two different shRNAs, acting on different signaling pathways in endothelial cells. One is targeting CD105 and VEGF-dependent pathway, and the other CD146 and VEGF-independent pathway. Consequently, simultaneously silencing two main pathways makes this plasmid potentially more efficient than others previously used. A delivery method used, GET, has already proven effective also in clinics, and by combining this therapy with IR makes it tentative for further usage. By in vitro testing, we have demonstrated functional and therapeutic ability of a newly constructed plasmid, thus supporting in vivo testing.

## 4. Materials and Methods

### 4.1. Construction of Plasmids

**Scheme 1:**
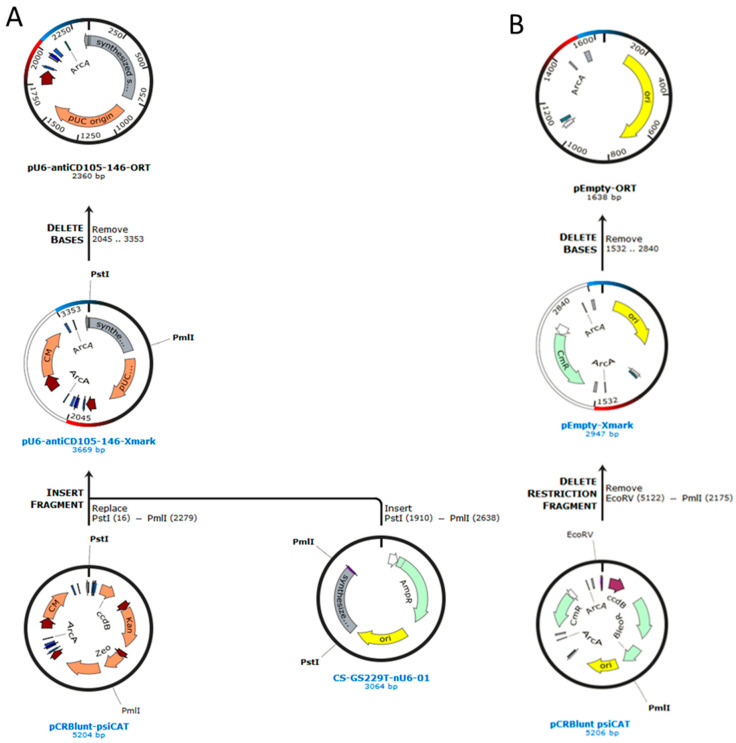
Construction of plasmids. (**A**) Cloning plan for the construction of pU6-antiCD105-146-ORT and (**B**) pEmpty-ORT plasmid.

Conventional molecular cloning techniques of restriction and ligation followed by transformation into competent *Escherichia coli* cells were used for the construction of plasmids. All the reagents and kits used for cloning (GeneJET Plasmid Miniprep Kit, TransformAid Bacterial Transformation kit supplied with *E. coli* strain JM109, Fast digest restriction enzymes, Rapid DNA Ligation Kit, GeneJET Gel Extraction Kit, SybrSafe) were purchased from Thermo Scientific (Waltham, MA, USA). Artificial gene synthesis service (GeneCopoeia, Rockville, MD, USA) was used to synthesize the expression cassette. ORT technology using DH1-PEPA and DH1-ORT *E. coli* cells (Cobra Biologics, Memphis, TN, USA) was used to prepare the final antibiotic free plasmid. SnapGene software was used for the sequence assembly, cloning planning and simulation of agarose gel electrophoresis. The expression cassette sequence for pU6-antiCD105-146-ORT plasmid was prepared by assembling the sequence for CD105 shRNA from pENTR/U6 CD105 plasmid [11] and sequence for CD146 from pMCAM [16]. Each sequence was placed under its own U6 promoter and desired restriction sites were added at each end of the expression cassette for future cloning. The sequence was ordered in a vector with ampicillin resistance, CS-GS229T-nU6-01. The vector was transformed in JM109 *E. coli* cells, amplified in bacteria and isolated by plasmid miniprep kit. The expression cassette was cut out with *PmlI* and *PstI* restriction enzymes, extracted from the electrophoretic gel and directionally cloned in pCRBlunt-psiCAT vector (Cobra Biologics). The resulting recombinant plasmid with the desired expression cassette, but still containing the antibiotic resistance gene, was transformed into the DH1-PEPA *E. coli* cells (Cobra Biologics) and transformed bacteria were selected on selective LB agar plates with chloramphenicol (Merck, Kenilworth, New Jersey, NJ, USA). The plasmid was isolated from DH1-PEPA cells and transformed into the DH1-ORT *E. coli* cells (Cobra Biologics), in which the antibiotic resistance gene was excised by *Xer* recombination. Transformation was followed by selection of a clone that contained the antibiotic free version of the plasmid and gave the highest yields according to miniprep isolation (Scheme 1A). The empty control plasmid pEmpty-ORT was prepared by blunt end restriction of the pCRBlunt-psiCAT vector with *PmlI, EcoRV* and back ligation followed first by transformation into DH1-PEPA and then to DH1-ORT *E. coli* cells (Scheme 1B). The successful construction of pU6-antiCD105-CD146-ORT and pEmpty-ORT, was confirmed by restriction analysis and full-length plasmid sequencing (Applied Biological Materials, Richmond, Canada). Presence of possible CpG islands was tested using an online tool DataBase of CpG islands and Analytical Tool (DBCAT) (http://dbcat.cgm.ntu.edu.tw. Accessed on 23 February 2021).

### 4.2. Purification of Transfection-Grade Plasmids

For the transfection experiments, plasmids were isolated and purified using the EndoFree Plasmid Mega Kit (Qiagen, Hilden, Germany) and diluted in endotoxin-free water to a concentration of 1 mg/mL. Plasmid concentrations were determined spectrophotometrically (Epoch Microplate Spectrophotometer, Take3™ Micro-Volume Plate, BioTek, Bad Friedrichshall, Germany). Additionally, plasmids quality was confirmed by 260/280 ratio and by restriction analysis.

### 4.3. Cells

Mouse endothelial cell line 2H-11 (ATCC^®^ CRL-2163™) and mouse skin melanoma B16 F10 (ATCC^®^ CRL-6475™) were purchased from American Type Culture Collection (ATCC, Manassas, VA, USA). The cells were cultured in advanced Dulbecco’s modified Eagle’s medium (DMEM, Gibco, Thermo Fisher Scientific, Waltham, MA, USA) supplemented with 5% fetal bovine serum (FBS, Gibco), 10 mM L-glutamine (Gibco), 50 µg/mL gentamicin (Gibco) and 100 U/mL crystacillin, at 37 °C in a humidified incubator with 5% CO_2_. Cells were regularly tested for the presence of Mycoplasma with MycoAlertTM PLUS Mycoplasma Detection kit (Lonza, Basel, Switzerland) and found to be negative.

### 4.4. Gene Electrotransfer

A monolayer of 80% confluent cell culture was trypsinized and washed with the corresponding supplemented medium. After 5 min centrifugation, the cell pellet was washed in ice-cold electroporation buffer (EP buffer: 125 mM sucrose; 10 mM K_2_HPO_4_, 2.5 mM KH_2_PO_4_ and 2 mM MgCl_2_ × 6H_2_O), centrifuged and resuspended at a concentration of 2.5 × 10^7^ cells/mL. The 40 µL of cell suspension (1 × 10^6^ cells) was mixed with 10 µL (1 µg/µL) of plasmid, and 50 µL of mixture was immediately pipetted between two stainless steel plate electrodes 2 mm apart and electric pulses were applied by the electric pulse generator GT-01 (Faculty of Electrical Engineering, University of Ljubljana, Slovenia). Electric pulse parameters were: 8 square-wave electric pulses of amplitude 120 V (amplitude over distance ratio 600 V/cm), pulse duration 5 ms and repetition frequency 1 Hz). After the electroporation, the cells were incubated for 5 min at room temperature with 100 µL of FBS and plated for following assays.

### 4.5. Irradiation

**Scheme 2:**
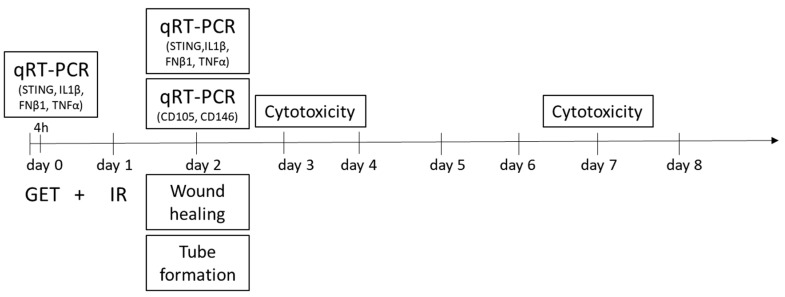
Study design. Timeline of experiments. GET—gene electrotransfer, IR—irradiation. Gene electrotransfer was performed on day 0, and 24 h later, on day 1, the cells were irradiated. The silencing of the targeted genes (CD105 and CD146) was evaluated on day 2, 48 h after GET, by qRT-PCR. On the same day, biological properties of the treated cells were evaluated by wound healing assay, where cells were plated and the next day, after the culture slide insert removal, the migration rate was evaluated. Tube formation assay was performed on day 2, where tubular complexes were formed 2.5 h after the cells were plated. On the same day, 24 h after the irradiation, the induction of *Sting*, *Ilβ1*, *Ifn-β1* and *Tnf-α* was quantified by qRT-PCR. The cytotoxicity assay was performed on day 3 and 7 after GET.

A dose of 5 Gy, at the dose rate of 1.8 Gy/min, was delivered to the cells 24 h after gene electrotransfer (Scheme 2). Glumay MP1-CP225 X-Ray Generator (Gulmay Medical Ltd., Byfleet, UK) operating at 200 kV, and 9.2 mA with Cu (0.55 mm) and Al (1.8 mm) filtering was used for irradiation of cells.

### 4.6. Cytotoxicity Assay

After GET, cells were plated in transparent flat bottom 96-well plates (Corning Incorporated, Corning, NY, USA) in the supplemented medium. After 24 h, some of the groups were irradiated as described above. On day 3 and day 7 (Scheme 2), the cytotoxicity was evaluated by adding 10 µL of PrestoBlue (Thermo Fisher Scientific, USA) in each well and 1 h after fluorescence intensity (excitation 560 nm and emission 590 nm) was measured by the microplate reader (Infinite 200, Tecan, Männedorf, Switzerland). The viability of the cells after treatment was normalized to the viability of control untreated cells.

### 4.7. Tube Formation Assay

**Scheme 3:**
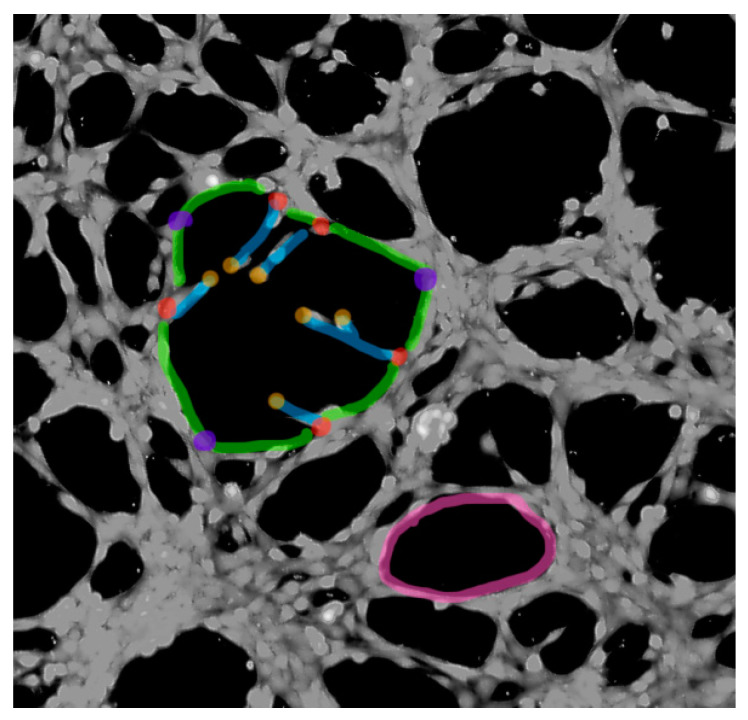
Endothelial tube formation analysis FIJI Angiogenesis Analyzer. Marked are mashes (magenta), nodes (red), junctions (violet), extremities (orange), branches (blue) and segments (green).

Tube formation assay was performed to determine the effect of the treatments on the ability of 2H-11 cells to form capillary-like structures. The cells (1.5 × 10^4^ per well) were plated, 48 h after the GET and 24h after IR (Scheme 2), on a μ-Slide Angiogenesis (Ibidi GmbH, Gräfelfing, Germany) covered with a BD Matrigel Basement Membrane Matrix, Phenol Red Free (BD Bio-sciences, San Jose, CA, USA) and incubated for 2.5 h until the formation of tubular complexes. The tubular complexes were stained with Calcein AM (Sigma-Aldrich, St. Louis, MO, USA). Images were captured with a digital camera (DP72, Olympus) connected to inverted fluorescent microscope (IX70, Olympus). Each image was further analyzed with FIJI image analyzer tool Angiogenesis Analyzer to determine the number of mashes, nodes and junctions, and total branching and segment length [77]. Nodes are defined as pixels having at least three neighbors; junction as groups of nodes forming a bifurcation; segments as binary lines linking two junctions; branches as binary lines linking one junction and one extremity and mashes as closed areas surrounded by segments (Scheme 3).

The values obtained after treatment were normalized to the values in the control untreated cells.

### 4.8. Wound-Healing Assay

Wound-healing assay was performed on the endothelial 2H-11 cells to determine the antimigratory effects of the treatments. The cells (1.7 × 10^4^ per well) were plated, 24 h after the IR and 48 h after the GET (Scheme 2), on a 24-well plate with silicone inserts forming a 500 μm ± 50 μm cell-free gap (24 Culture-Inserts, Ibidi, Munich, Germany). After 24 h incubation, a confluent monolayer was formed and the culture inserts were removed using sterile tweezers. In each well, a 1 mL of supplemented medium was added and the wells were observed under the inverted microscope (Olympus IX-70, Germany), connected with a digital camera (DP72, Olympus). The images of the wells were taken at time of insert removal and every 2 h thereafter until the gap was closed. From the images, the cell-free area was quantified in a FIJI image analysis at all-time points, which provided values for kinetic analysis, as described previously [10]. The values obtained after treatment were normalized to the values in the control untreated cells.

### 4.9. Quantitative Real-Time PCR

Quantitative real-time PCR was performed 48 h after GET to determine silencing of CD105 and CD146. Additionally, to determine expression of DNA sensor *Sting* and cytokines *Il1β*, *Ifn-β1* and *Tnf-α*, quantitative real-time PCR was performed 4 h after GET and 24 h after IR of 2H-11 cell line (Scheme 2). Cell pellets were collected and the total RNA was extracted from the cells with peqGOLD total RNA kit (VWR, Langenfeld, Germany). Concentrations of RNA were quantified by a spectrophotometer at 260 nm (Epoch). The purity of RNA was determined by measuring the ratio of absorbance at 260 nm/280 nm. RNA was reverse transcribed into complementary DNA (cDNA) using a SuperScript VILO cDNA Synthesis Kit (ThermoFisher Scientific, USA). Diluted mixtures were used as a template for the qRT-PCR. Based on our previous optimization TaqMan chemistry was used to detect CD105 and CD146 [10] and Syber green chemistry to detect *Sting*, *Il1β*, *Ifn-β1* and *Tnf-α* [56]. To determine the gene expression of CD105 and CD146 Taqman Gene Expression Assay containing a pair of primers and TaqMan probes to amplify the fragments of murine Mcam cDNA (Mm00522397_m1) and endoglin cDNA (Mm00468256_m1). As an internal control TaqMan probes were used to amplify GAPDH (Mm99999915_g1). To determine gene expression of *Sting*, *Il1β*, *Ifn-β1* and *Tnf-α* Syber Green method was used (PowerUP SYBER Green Master Mix, Appliedbiosystem) using the corresponding primers and cycling conditions (Supplementary Table S1 describing qRT-PCR oligonucleotides and Supplementary Table S2 providing qRT-PCR cycling conditions).

The qRT-PCR products were analyzed using Quant Studio Design and Analysis Software (Thermo Fisher Scientific). Relative quantification was performed by comparison to the housekeeping genes β-actin and glyceraldehyde 3-phosphate dehydrogenase (GAPDH) using the ΔΔCt method. Expression of *Sting*, *Il1β*, *Ifn-β1* and *Tnf-α* was presented as fold change in expression compared to control. Silencing was presented as the percentage of expression in control. Additionally, the levels of CD105 and CD146 mRNA expression in endothelial cells were presented as the threshold cycle value (Ct).

### 4.10. Statistical Analysis

GraphPad Prism version 8.1.2. (GraphPad Software, San Diego, CA, USA) was used for statistical analysis and graphical representations of the results. All data were tested for normality using the Shapiro–Wilk test. Data are presented as the arithmetic mean (AM) ± the standard error of the mean (SE). One-way ANOVA followed by the Holm–Sidak test for multiple comparisons was used for the determination of significant differences (*p* < 0.05) between groups. In the silencing experiments, an unpaired *t*-test was used to determine the significant differences (*p* < 0.05).

## Data Availability

The presented data is available upon request.

## Supplementary Materials

The following are available online at https://www.mdpi.com/1422-0067/22/6/3069/s1: Supplementary sequence S1: pU6-antiCD105-146-ORT full sequence, Supplementary sequence S2: pEmpty-ORT full sequence, Table S1: qRT-PCR oligonucleotides, Table S2: qRT-PCR cycling conditions.

## Abbreviations

CD105	Endoglin
CD146	Mcam
DAMP	Danger Associated Molecular Pattern
GAPDH	Glyceraldehyde 3-phosphate dehydrogenase
GET	Gene electrotransfer
IFN-β1	Interferon β1
IL1β	Interleukin 1-β
IR	Irradiation
ORT	Operator repressor titration
PAMP	Pathogen Associated Molecular Pattern
qRT-PCR	Quantitative reverse transcription polymerase chain reaction
shRNA	Short hairpin RNA
TGF-β	Transforming growth factor β
TNF-α	Tumor Necrosis Factor alpha
VEGF	Vascular endothelial growth factor
