# Supplementary material for: Evaluation of a Novel Plasmid for Simultaneous Gene Electrotransfer-Mediated Silencing of CD105 and CD146 in Combination with Irradiation
Source: Int J Mol Sci. 2021 Mar 17;22(6):3069. doi: 10.3390/ijms22063069 (PMC8002395; doi:10.3390/ijms22063069)
Supplement: Supplementary file 1 [file ijms-22-03069-s001.pdf]

Supplementary sequence S1: pU6-antiCD105-146-ORT full sequence

AGGGCGAATTCTGCAGATAACCAATTCAGTCGACTGGATCCGGTACCAAGGTCGGGCAGGAAGAGGGCCTAT
TTCCCATGATTCCTTCATATTTGCATATACGATACAAGGCTGTTAGAGAGATAATTAGAATTAATTTGACTGTAA
ACACAAAGATATTAGTACAAAATACGTGACGTAGAAAGTAATAATTTCTTGGGTAGTTTGCAGTTTTAAAATTA
TGTTTTAAAATGGACTATCATATGCTTACCGTAACTTGAAAGTATTTCGATTTCTTGGCTTTATATATCTTGTGGA
AAGGACGAAACACCGCCTGCTGTTTGTCATCTATAGCTTTCGAAAAAGCTATAGATGACAAACAGCAGGTTTT
GCTAGCGCTACCGGACTCAGATCTCGAGCTCAAGCTTCGAATTAAGGTCGGGCAGGAAGAGGGCCTATTTCCC
ATGATTCCTTCATATTTGCATATACGATACAAGGCTGTTAGAGAGATAATTAGAATTAATTTGACTGTAAACAC
AAAGATATTAGTACAAAATACGTGACGTAGAAAGTAATAATTTCTTGGGTAGTTTGCAGTTTTAAAATTATGTT
TTAAAATGGACTATCATATGCTTACCGTAACTTGAAAGTATTTCGATTTCTTGGCTTTATATATCTTGTGGAAAG
GACGAAACACCGCCAAACTGGTGTGCGTCTTCTTGTTCGAAAACAAGAAGACGCACACCAGTTTGGTTTTTCA
CACGTGCTAAAACTTCATTTTTAATTTAAAAGGATCTAGGTGAAGATCCTTTTTGATAATCTCATGACCAAAATC
CCTTAACGTGAGTTTTCGTTCCACTGAGCGTCAGACCCCGTAGAAAAGATCAAAGGATCTTCTTGAGATCCTTT
TTTTCTGCGCGTAATCTGCTGCTTGCAAACAAAAAAACCACCGCTACCAGCGGTGGTTTGTTTGCCGGATCAAG
AGCTACCAACTCTTTTTCCGAAGGTAACTGGCTTCAGCAGAGCGCAGATACCAAATACTGTTCTTCTAGTGTAG
CCGTAGTTAGGCCACCACTTCAAGAACTCTGTAGCACCGCCTACATACCTCGCTCTGCTAATCCTGTTACCAGTG
GCTGCTGCCAGTGGCGATAAGTCGTGTCTTACCGGGTTGGACTCAAGACGATAGTTACCGGATAAGGCGCAG
CGGTCGGGCTGAACGGGGGGTTCGTGCACACAGCCCAGCTTGGAGCGAACGACCTACACCGAACTGAGATAC
CTACAGCGTGAGCTATGAGAAAGCGCCACGCTTCCCGAAGGGAGAAAGGCGGACAGGTATCCGGTAAGCGG
CAGGGTCGGAACAGGAGAGCGCACGAGGGAGCTTCCAGGGGGAAACGCCTGGTATCTTTATAGTCCTGTCGG
GTTTCGCCACCTCTGACTTGAGCGTCGATTTTTGTGATGCTCGTCAGGGGGGCGGAGCCTATGGAAAAACGCC
AGCAACGCGGCCTTTTTACGGTTCCTGGCCTTTTGCTGGCCTTTTGCTCACATGTTCTTTCCTGCGTTATCCCCTG
ATTCTGTGGATAACCGTATTACCGCCTTTGAGTGAGCTGATACCGCTCGCCGCAGCCGAACGACCGAGCGCAG
CGAGTCAGTGAGCGAGGAAGCGGAAGAGCGCCCAATACGCAAACCGCCTCTCCCCGCGCGTTGGCCGATTCA
TTAATGCAGCTGGCACGACAGGTTTCCCGACTGGAAAGCGGGCAGTGAGCGCAACGCAATTAATGTGAGTTA
GCTCACTCATTAGGCACCCCAGGCTTTACACTTTATGCTTCCGGCTCGTATGTTGTGTGGAATTGTGAGCGGAT
AACAATTTCACACAGGAAACAGCTATGACCATGATTACGCCAAGCTATTTAGGTGACACTATAGAATACTCAAG
CTATGCATCAAGCTTGGTACCGAGCTCGGATCCACTAGTAACGGCCGCCAGTGTGCTGGAATTCGCCCTTATAA
TGCTAGCGGTCATGAAGCTTATCATCGATAAGCTCATAGACAGCCTGAAACAGGCGATGCTGCTTATCGAATC
AAAGCTGCCGACAACACGGGAGCCAGTGACGCCTCCCGTGGGGAAAAAATCATGGCAATTCTGGAAGAAATA
GCGCTTTCAGCCGGCAAACCGGCTGAAGCCGGATCTGCGATTCTGATAACAAACTAGCAACACCAGAACAGCC
CGTTTGCGGGCAGCAAAACCCGTACTTTTGGACGTTCCGGCGGTTTTTTGTGGCGAGTGGTGTTCGGGCGGTG
CGCGCAAGATCCATTATGTTAAACGGGCGAGTTTACATCTCAAAACCGCCCGCTTAACACCATTCATGAGGCCG
CTAGCATTATA

Supplementary sequence S2: pEmpty-ORT full sequence

GTGCTAAAACTTCATTTTTAATTTAAAAGGATCTAGGTGAAGATCCTTTTTGATAATCTCATGACCAAAATCCCT
TAACGTGAGTTTTCGTTCCACTGAGCGTCAGACCCCGTAGAAAAGATCAAAGGATCTTCTTGAGATCCTTTTTTT
CTGCGCGTAATCTGCTGCTTGCAAACAAAAAAACCACCGCTACCAGCGGTGGTTTGTTTGCCGGATCAAGAGC
TACCAACTCTTTTTCCGAAGGTAACTGGCTTCAGCAGAGCGCAGATACCAAATACTGTTCTTCTAGTGTAGCCG
TAGTTAGGCCACCACTTCAAGAACTCTGTAGCACCGCCTACATACCTCGCTCTGCTAATCCTGTTACCAGTGGCT
GCTGCCAGTGGCGATAAGTCGTGTCTTACCGGGTTGGACTCAAGACGATAGTTACCGGATAAGGCGCAGCGG
TCGGGCTGAACGGGGGGTTCGTGCACACAGCCCAGCTTGGAGCGAACGACCTACACCGAACTGAGATACCTA
CAGCGTGAGCTATGAGAAAGCGCCACGCTTCCCGAAGGGAGAAAGGCGGACAGGTATCCGGTAAGCGGCAG
GGTCGGAACAGGAGAGCGCACGAGGGAGCTTCCAGGGGGAAACGCCTGGTATCTTTATAGTCCTGTCGGGTT
TCGCCACCTCTGACTTGAGCGTCGATTTTTGTGATGCTCGTCAGGGGGGCGGAGCCTATGGAAAAACGCCAGC

AACGCGGCCTTTTTACGGTTCCTGGCCTTTTGCTGGCCTTTTGCTCACATGTTCTTTCCTGCGTTATCCCCTGATT
CTGTGGATAACCGTATTACCGCCTTTGAGTGAGCTGATACCGCTCGCCGCAGCCGAACGACCGAGCGCAGCGA
GTCAGTGAGCGAGGAAGCGGAAGAGCGCCCAATACGCAAACCGCCTCTCCCCGCGCGTTGGCCGATTCATTA
ATGCAGCTGGCACGACAGGTTTCCCGACTGGAAAGCGGGCAGTGAGCGCAACGCAATTAATGTGAGTTAGCT
CACTCATTAGGCACCCCAGGCTTTACACTTTATGCTTCCGGCTCGTATGTTGTGTGGAATTGTGAGCGGATAAC
AATTTCACACAGGAAACAGCTATGACCATGATTACGCCAAGCTATTTAGGTGACACTATAGAATACTCAAGCTA
TGCATCAAGCTTGGTACCGAGCTCGGATCCACTAGTAACGGCCGCCAGTGTGCTGGAATTCGCCCTTATAATGC
TAGCGGTCATGAAGCTTATCATCGATAAGCTCATAGACAGCCTGAAACAGGCGATGCTGCTTATCGAATCAAA
GCTGCCGACAACACGGGAGCCAGTGACGCCTCCCGTGGGGAAAAAATCATGGCAATTCTGGAAGAAATAGCG
CTTTCAGCCGGCAAACCGGCTGAAGCCGGATCTGCGATTCTGATAACAAACTAGCAACACCAGAACAGCCCGT
TTGCGGGCAGCAAAACCCGTACTTTTGGACGTTCCGGCGGTTTTTTGTGGCGAGTGGTGTTCGGGCGGTGCGC
GCAAGATCCATTATGTTAAACGGGCGAGTTTACATCTCAAAACCGCCCGCTTAACACCATTCATGAGGCCGCTA
GCATTATAAGGGCGAATTCTGCAGAT

Supplementary table S3. qRT-PCR oligonukleotides

	Oligonucleotides (IDT, USA)	Primer sequence
STING	mSTINGF	GTCCTCTATAAGTCCCTAAGCATG
	mSTINGR	AAGATCAACCGCAAGTACCC
IL1β	IL1b-140f	AGTTGACGGACCCCAAAAGA
	IL1b-232r	TGCTGCTGCGAGATTTGAAG
IFNβ1	IFNb1-241F	TGCCATCCAAGAGATGCTCCAGAA
	IFNb1-364R	AGAAACACTGTCTGCTGGTGGAGT
TNFα	TNFαF	CCCTCCAGAAAAGACACCATG
	TNFαR	GTCTGGGCCATAGAACTGATG

Supplementary table S4. qRT-PCR cycling conditions using a qRT-PCR Kit - Syber Select Master Mix (Thermo Fisher scientific)

Number of cycles	Temperature	Time
1	50 °C	2 min
	95 °C	2 min
40	95 °C	15 sec
	60 °C	15 sec
1	72 °C	1 min
